# State Regulation, Family Breakdown, and Lone Motherhood: The Hidden Costs of World War I in Scotland

**DOI:** 10.1177/0363199014548826

**Published:** 2014-10

**Authors:** Annmarie Hughes, Jeff Meek

**Affiliations:** 1 University of Glasgow, Glasgow, UK

**Keywords:** war, regulation, lone mothers, family breakdown, Scotland

## Abstract

Using a range of parish records, records from the Registrar General of Scotland, charity organizations, and media reports, this article contributes to the historiography which evaluates the effects of World War I in Britain as well as the history of lone mothers and their children. It highlights how during the war, women, especially lone mothers, made significant gains through the welfare system, changing approaches to illegitimacy and the plentiful nature of women’s work but also how in doing so this brought them under greater surveillance by the state, local parishes, and charity organizations. Moreover, as this article will demonstrate, many of the gains made by women were short-lived and in fact the war contributed to high levels of family breakdown and gendered and intergenerational poverty endured by lone mothers and their children.

In 1919, the Executive Committee of the Royal Scottish National Society for the Prevention of Cruelty to Children (SNSPCC) expressed anxiety over what they saw as the effects of World War I on marriage and the family. They stated that “one feature of modern life that has engaged us during the past year is that there has been a tendency towards the breakup of the home life.” By this, they were referring to the destitution experienced by women and children as ex-servicemen increasingly deserted their families.^1^ Thane highlights how there were many routes into lone motherhood between the wars and these included not only unmarried motherhood, but also desertion, divorce, separation, and widowhood.^2^ World War I contributed to a rise in the number of lone parents in Scotland in all of these forms. The Scottish Census of 1921 accessed the number of “fatherless” children in Scotland, highlighting concerns over the impact of war on family life. What the census demonstrates is that in addition to those children living in single-parent households due to desertion, wartime rises in illegitimacy and higher rates of divorce, almost one-third of all Scottish children were living with one parent because of widowhood.^3^ The experiences of lone parents and children and on family life in Britain wrought by World War I remain relatively underdeveloped. With the centenary of outbreak of the conflict approaching, the history of the war continues to be dominated by military history and considerations of the economic aftermath of war.^4^ In Scottish history, this is particularly marked, and the focus of wartime studies concentrates largely on the economic consequence of the war, the industrial and community militancy that occurred during the war, and the subsequent progress of the Independent Labor Party in Scotland.^5^

Although there has been more historical attention paid to the gendered, demographic, and social implications of war, it remains the case that the history of women’s experiences during World War I has mainly been evaluated through the prism of their entry into what had been considered men’s jobs and in their role and efforts in the medical profession during the conflict.^6^ Historians have also questioned whether women gained a greater measure of equality because of their wartime contribution or, alternatively, faced a male backlash in the postwar attempts to return to normalcy.^7^ Dilution schemes did facilitate some women’s entry into traditional masculine jobs, although generally on unequal terms with men. Women also penetrated jobs in commerce, retail, and transport during the war while others were employed in well-paid munitions work, particularly young women. These studies, while important contributions to our understanding of gender during wartime conditions, reveal very little about the effects of war on women as mothers and wives or the impact of war on family life.

Pedersen highlights how the wartime extension of work for women benefited many married women too, especially those who had endured their husbands’ abuses of the family wage or their irregular and low paid wages.^8^ She also evaluates the economic impact of World War I on the wives and the children of servicemen through the lens of the agitation for separation allowances paid by the state to those whose husbands were on active service and also the social and political implications for the wives in receipt of these benefits. Separation allowances were “structured around maintaining the domestic rights of men” and this led to state regulation over the wives of soldiers. A wife’s eligibility for a separation allowance was based on a husband’s status as a soldier rather than her own contribution to wartime society. As such, separation allowances could be used to justify the state, in the form of the Ministry of Pensions, taking on the role of a surrogate husband morally and financially. Control over soldiers’ wives sexual and social conduct was imposed as a condition of eligibility for separation allowances.^9^ Lomas illustrates how the regulation of women’s sexual and social behavior was also imposed on the widows of servicemen who received war pensions. She argues that prewar negative stereotypes about working-class women ensured that the benefit system was framed in such a way so as to guarantee the “good behavior” of widows.^10^ Single women and lone mothers also attracted attention because fear over unregulated female sexuality and illegitimacy brought them under the scrutiny of the agencies of the state.^11^ However, as this article will highlight, in Scotland, it was not only the surveillance conducted by the Ministry of Pensions which soldiers’ wives, widows, lone mothers, and young working-class women had to contend with but also that effected by parish councils, charity organizations, the media, and working-class men.

This article uses a range of official sources, parish council records, the records of charity organizations, and newspaper reports to assess the effects of World War I on women and children demonstrating how, in Scotland, World War I resulted in greater state regulation of single and married women. The war destabilized gender roles, and this resulted in fears over female sexuality in the absence of husbands and fathers. The article also discusses the relationship between state agencies and the wives of men on active service, of war widows, and of lone mothers. Some women benefited from the conditions of war but in the postwar years, as a consequence of war and the adverse postwar economic climate in Scotland, there was an increase in family breakdown resulting in a greater number of female-headed households and therefore gendered and intergenerational poverty that affected women and children.

## Separation Allowances, Soldiers’ Wives, and State Regulation

As was the case throughout Britain, World War I expanded employment opportunities for women including those who were widowed, deserted, and separated and women whose husbands were on active service. Much of the work was relatively well paid.^12^ Wives of servicemen who proved themselves eligible were also entitled to the financial protection of the state in the form of separation allowances. For poorer women, especially those whose husbands had had irregular or low incomes or who had abused the family wage, separation allowances offered “economic security, independence, and dignity.” Pedersen details how the wife of an agricultural worker with four children was financially better off with a separation allowance than living off of her spouse’s wage. Moreover, women married to unskilled workers were not disadvantaged either because the allowance was equal to the sum of money husbands gave their wives after they had made their own personal deductions. Wives with older children also benefited from the allowance and the availability wages from their offspring.^13^ Some women who were in receipt of a separation allowance which was less than their husbands’ prewar wages were assisted by their spouses’ peacetime employers who paid them a pension to make up the shortfall. This was the policy of the SNSPCC who provided the wives of their serving officers with a pension to make up the difference between their separation allowances and their husbands’ wage.^14^ Indeed, the SNSPCC’s executive lamented that it was “sad to think that in the absence of fathers the home is in many cases better off.” Separation allowances and regular work and the absence of husbands who put personal claims, sometimes excessively, on family resources meant that children were “better fed” and the family’s standard of living improved.^15^

However, not all wives benefited financially from separation allowances because they were subsistence payments and not all prewar employers paid pensions. The income that wives would have received through separation allowances was insufficient to entice skilled workers to enlist in the early years of the war in Scotland. In 1914, a wife with four children was entitled to 17 shillings 6 pence when the wages of a coal miner were 31 shillings 9 pence. Allowances did increase and by 1918, a mother of four children was in receipt of 31 shillings but by this point coal miners were earning 60 shillings 2 pence and fitters and turners 67 shillings 4 pence.^16^ Thus, separation allowances had to be raised to encourage married men in skilled occupations to enlist in the services because the men’s primary concern was whether their families would be financially secure if they enlisted or were killed on active service.^17^ Moreover, during the first two years of the war, women experienced long delays in receiving allowances because the state’s apparatus could not cope with demand.^18^ This was at the point where inflation was reducing real wages and separation allowances did not keep pace with inflation until at least 1917.

Over time separation allowances did safeguard the living standards of many soldiers’ families, but they also increased the state’s ability to monitor the behavior of soldiers’ wives. The benefits were not a wife’s in her own right and this facilitated the state placing conditions on the receipt of benefits. Separation allowances could be withdrawn for “immorality, conviction of a criminal charge, gross neglect of children, lack of cleanliness and drunkenness.”^19^ Authorities also informed husbands of their wives’ indiscretions and allowed the men to decide whether their wives “deserved” to continue to receive an allowance. These attitudes had underpinned the surveillance of working-class women in Scotland who were in receipt of parish relief and charity well before the introduction of separation allowances. For example, before the war it had been the policy of Glasgow Parish Council to withdraw widows’ outdoor relief for “drunken and immoral behavior” and keeping “dirty houses.”^20^

Throughout the war, working-class wives were treated with suspicion and subject to a demonizing rhetoric about their behavior and if found wanting they too were often harshly treated. It was a widely circulated opinion that some soldiers’ wives were squandering separation allowances on alcohol and frivolity while their husbands fought in the war. From the onset of war, officials of Glasgow Parish Council were of the opinion that there had been an increase in alcohol consumption and child neglect among soldiers’ wives. Somewhat undermining his own argument, James R. Motion, Glasgow’s Inspector for the Poor, insisted that as well as the considerable attention being given to soldiers’ wives by Scottish Parish Councils, a large number of cases of drunkenness and child neglect and “filthy and wretched homes” had been uncovered by investigations conducted by SNSPCC, the Soldiers and Sailors Family Association, and other charitable organizations which might “not otherwise have been brought to light.”^21^ However, as the SNSPCC’s officers pointed out that some of the women under their gaze had consumed alcohol before the war. The Society also believed that alcohol consumption was a short-term manifestation of women receiving large sums of money in back payments because of the delays they had experienced in receiving their separation allowances. Apparently after this initial windfall, it did not take long for most wives to “straighten themselves out.”^22^

The SNSPCC took a more paternalistic view claiming that much of the drunkenness among soldiers wives was due to “well-founded fears and anxiety over their husbands being called up” and also because of the “readjustment of the family life” in which women had to take on sole responsibility for the well-being of the family unit.^23^ Nonetheless by 1915, the views of charity and parish officials on soldiers’ wives squandering separation allowances and neglecting their children through drunkenness were pervading the pages of the Scottish media. The *Scotsman* reported on how Glasgow Parish Council had provided evidence for thirty-five successful prosecutions against the wives of soldiers who had been charged under the Children’s Act because they had neglected their children. Identified as coming from “a very low class” and living “mostly in slums,” the women were also accused of being habitual drunks and immoral.^24^ Seemingly in one tenement close alone, four soldiers’ wives were neglecting their children and having a “regular carousel every Monday when they got their allowances.”^25^ The root of the problem according to Motion was “that in practically every case of drunkenness and child neglect,” the soldiers’ wives had more money because they have larger families and therefore “bigger separation allowances.” He went on, “bereft of their husbands company many wives take up the company of undesirable neighbours who do all in their power to help them spend their separation allowances.” What the media failed to report, however, was that Motion knew full well and had acknowledged that the majority of soldiers’ wives did not conduct themselves in this manner.^26^

For all the anxiety over the women’s behavior, less than 2 percent of British wives lost their separation allowance due to their “misconduct.” Pedersen argues that although few women lost their allowance, those who did were exemplars of what misconduct could bring, acting as a deterrent to other women.^27^ Few women may have lost their separation allowances, but the discourse on the misconduct of soldiers wives led to greater policing of women and this increased the number of women and their children who came to the attention of Scottish Parish Councils. If the women who were being investigated were found to be under the influence of alcohol, this all but ensured a prosecution.^28^ The records for Glasgow Parish Council show that over eighty soldiers’ wives were prosecuted for child neglect, mainly failure to supervise their children or because the children were ill clad, ill fed, or the home lacked furnishings and bedding.^29^

During the war, the wives prosecuted by Glasgow Parish Council for child neglect would have forfeited their separation allowance, but also in many instances their children too. Other wives had the neglect charges against them dropped if they agreed to have their children placed in care until the children were sixteen years of age or at a later date the mother could prove to a court that she had mended her ways. In 1915, Glasgow Parish Council investigated 321 cases of child neglect involving 1068 children. Of these, 149 cases involving 514 children were against soldiers’ wives. Many were cautioned, but forty-two wives were taken to court and prosecuted. The parish insisted that the SNSPCC and the Sanitary Board had dealt with similar complaints and if these were tabulated together, the seriousness of soldiers’ wives conduct would be much more apparent.^30^

There were women who neglected their children, and some did so because of their alcohol consumption, but when officials evaluated how well fed and clothed children appeared and how well furnished and cared for homes were, they ignored the long-term effects of the Scottish economy: between 1903 and 1910, there had been three severe recessions which decimated regional economies. There was a further economic downturn of 1913.^31^ This was followed by the inflationary pressures of the early years of the war on food, clothing, and rent prices—which in 1915 led to community militancy in the form of rent strikes in Scotland.^32^ Coetzee insists that many families’ finances became untenable by the winter of 1914–1915 as net incomes failed to keep up with inflation. This would have been much worse for those on fixed incomes such as soldiers’ wives in receipt of separation allowances.^33^ Charity and parish officials also failed to recognize that what they considered a lack of cleanliness or “wretched and filthy” homes of the wives who resided in the slums were partly a reflection of to the extremely poor housing conditions for which cities like Glasgow were renowned and in which women fought a losing battle against dampness, infestation, and unsanitary conditions.^34^ Many soldiers’ wives had struggled against low and irregular wages and it took time for the economic benefits of separation allowances to be realized and translated into improved living standards. Moreover, the number of prosecutions for neglect by soldiers’ wives fell with improvements in the real value of separation allowances as incomes caught up with prices after 1917, but nevertheless across Scotland charity and parish officials took it upon themselves to visit, “counsel and advise” soldiers’ wives, and wives more generally, in household management, child care, and to monitor their conduct and initiate prosecutions against those women who failed to meet their standards.

They also tackled other concerns about the conduct of soldiers’ wives which apparently emerged, as living standards improved. In 1917, the SNSPCC argued that although “high wages” has meant that the physical condition of children had improved, they still insisted that malnutrition continued to plague children due to “ignorance and carelessness of mothers.” They also claimed that while there had been a reduction in the problem of excessive drinking which had caused child neglect, there had emerged a new form of neglect. Soldiers’ wives “whose husbands are on military service for a lengthened period of time … to relieve monotony and strain” were leaving their children unattended. They claimed to have some sympathy for the women who were “wearing anxiety and the sole responsibility for the family” and who gave into “the temptation of the cinema.” Some seemingly went out at night “after the day’s work” was completed leaving “younger children with the oldest, children of 11 and 12 years.”^35^

Much has been written thus far about the regulation of wives at the behest of external agencies, but local records also indicate that the desire to have wives’ behavior monitored was also instigated by husbands on active service. According to local officials, soldiers at the front were sending letters asking them to look after their children and for that matter their wives “whom they knew to have gone astray.”^36^ The state and state agencies may have acted as surrogates of husbands who were on active service to regulate the behavior of their wives, but this was not always a one-way process as there were men who expected them to do so in their absence.

## Single Women, Illegitimacy, and Surveillance

It was not just married women who came under greater surveillance. Among the first women to come under the lens of the state agencies were single women, especially, young working-class women. These young females were among the greatest beneficiaries of women’s wartime economic progress because they were the first to be enlisted to substitute for men who were called to arms and this gave them greater spending power to enjoy the modern leisure pursuits such as dance halls, cafés, cinema-going, and access to new fashions and makeup. Woollacott argues that young women became targets of regulation due to concerns over what the press would label “khaki fever.” This was the idea that young women, especially those who resided near army camps, were attracted by the excitement of war and to men in uniform which it was feared would result in high levels of illegitimacy, prostitution, and venereal disease.^37^ According to the Registrar General for Scotland in his Annual Report of 1916, there had been serious concerns that the war would lead to an increase in illegitimate births.^38^ This was not only due to khaki fever, but also related to the notion that departing or returning soldiers, uncertain of their futures, would engage in premarital sex.

These fears were translated into the establishment of women’s patrols across Britain.^39^ In Scotland, the patrols worked with the police, were sanctioned by the Scottish Office, and were run by the National Union of Women Workers.^40^ In 1915, there were 425 patrols in Scotland based in twenty different locations.^41^ Edinburgh had thirty women’s patrols by 1916.^42^ Women’s patrols monitored “girls” whose behavior was regarded as unsatisfactory, those who gathered in the vicinity of military camps, who might potentially tempt soldiers into misconduct.^43^ The *Scotsman* newspaper called the patrols “an organized chaperone system” to avert the development of “loose and dissolute habits” and increased levels of illegitimacy, especially as the men who were seen as most likely to succumb to the charms of the young women were older married men—so there would be no opportunity of averting illegitimacy through marriage.^44^ To this end, the Defense of the Realm Act was used to regulate women’s behavior. For example, in Cupar, Fife, there was a military curfew which forbade women from leaving their homes after 10 p.m. at night.^45^

Yet for all the fears expressed, illegitimacy did not increase significantly in Scotland during the war. Table 1 below highlights the number of illegitimate births peaked at nearly 9000 in 1912 before falling steadily to a low of 7,295 in 1917. However, the number of births in Scotland also fell during the war period. The average percentage of illegitimate births to total births in the period 1911–13 was 7.32, which remained unchanged for the war years.^46^ Nevertheless, more than 50,000 illegitimate children were born in Scotland between 1914 and 1919. 

**Table 1.** Number and Percentage of Illegitimate Births in Scotland.

Year	Number	Percentage of Births
1912	8,989	7.32
1913	8,548	7.10
1914	8,879	7.16
1915	7,875	6.90
1916	7,797	7.10
1917	7,295	7.49
1918	7,854	7.97
1919	8,424	7.93

*Source:* Registrar General of Scotland, Annual Reports, 1911–1920.

Indeed, according to the Registrar General of Scotland, at no time since the 1850s had illegitimacy rates been so low.^47^ The 1915 report stated,

The very general decline in the number of illegitimate births is noteworthy, because fears were expressed that conditions of war would lead to a marked increase of illegitimacy. It is satisfactory to record that the reverse is found to be the case.^48^

In England, the number of illegitimate births rose by 5 percent compared to rates in 1902.^49^ In Scotland, the rise was much smaller and was related to patterns of voluntary enlistment. Prewar illegitimacy rates were the highest in Scotland’s rural northeast and southeast where the highest rates of voluntary enlistment occurred. Before conscription was introduced, 68 percent of men of military age from Perth had enlisted for the army.^50^ Here, the illegitimacy rate was around 4 percent during the war; by contrast, in Kinross, just over 20 percent of men enlisted voluntarily and illegitimacy rates were around 15 percent.^51^ More than 50 percent of all the men of military age enlisted voluntarily between August 1914 and December 1916 from Scotland’s rural regions of Peebles, Midlothian, Sutherland, Inverness, Ross and Cromarty, and Elgin. However, voluntary enlistment was relatively high all over Scotland with between a third and a quarter of men enlisting voluntarily from the central belt. In the English, Welsh, and Irish counties, enlistment never rose above 50 percent and in most counties of England and Wales enlistment was between 20 percent and 30 percent. It was lower in Ireland where it ranged from 3.5 percent to 24 percent.^52^

In the nineteenth century, illegitimacy rates reached 25 percent of all births in some of Scotland’s rural regions and during the war they continued to be higher than in larger burghs where rates were around 6.25 percent as opposed to 8 percent in county districts.^53^ Nevertheless, the loss of young men to war did considerably reduce rural illegitimacy and mediated any increases that might have arisen across Scotland, particularly in instances where premarital pregnancy was not followed by marriage because of war deaths and service. However, not all illegitimate births were to single women outside stable relationships. Thane and Evans illustrate how the cost of divorce was prohibitive for poorer people and therefore many individuals separated and cohabited or bigamously married other people. These “illicit” or “irregular unions” were acknowledged by the state from 1916 in the provision of an “unmarried wife” separation allowance and war widows pensions which although having a more “stringent criteria” and lower remuneration nevertheless recognized in law “irregular unions” through the benefit system.^54^

It was unmarried mothers outside of stable unions who became a “public issue” during the war. Illegitimacy was attributed to loosening morals and a lack of parental supervision, especially as unmarried mothers could not apply for financial support from fathers in services lest they disrupt the war effort.^55^ Unmarried mothers were regarded as immoral and a drain on resources, but there was also anxiety over the “lost generation” of young men who had died in the conflict as well as over the high miscarriage rate and rising levels of infant mortality among unmarried mothers. Table 2 illustrates how, although fluctuating, by 1917 the infant mortality rate in Scotland was rising and would continue to do so in the interwar years but how it was almost double for illegitimate births in comparison with legitimate births. By 1921, it was double the level of legitimate births.^56^

**Table 2.** Infant Mortality for Illegitimate and Legitimate Births in Scotland, 1913–1917 per 1000 Live Births.

Year	Legitimate	Illegitimate
1913	96	152
1915	130	151
1917	113	212

*Source:* *Scotsman*, March 18, 1919.

This led to what the *Scotsman* newspaper labeled “the patriotic aspect of the problem” of illegitimacy.^57^ The loss of life during the war ensured that all babies became important and as a result child and maternal health provisions improved, including those for unmarried women and their children. Recognizing that many unmarried mothers remained in their own homes or in lodgings, the Glasgow Committee for the Care of Unmarried Mothers and their Children was established in 1917 to organize and cooperate in schemes to help unmarried mothers and their children. Involving the Medical Officer for Health, clerks to the parish councils, university settlements, the clergy, “social workers” from home and day nurseries and health visitors, the organization aimed to act as a “bureau of information and investigation” and to help unmarried mothers through systematic visitation and the provision of aftercare for mothers and children until the children reached the age where they could earn independently.^58^

One in ten babies born in Edinburgh, around 500, were illegitimate in 1919 when Edinburgh’s Public Health board began to consider establishing boarding houses for unmarried mothers where they could work by day and look after their babies when they returned home, thereby retaining their children.^59^ In Glasgow, the Women’s Help Committee, which had originally assisted deserted wives, took on the role of working with unmarried mothers after World War I. It acted as a branch of the National Council for Unmarried Mothers and Children which was established in England in 1918.^60^ Each year, the Committee dealt with up to 500 individuals who were identified as “delinquent girls.”^61^ Also acting as a pressure group for legislative change which would benefit unmarried mothers and their children, the Committee offered unmarried mothers advice, shelter, and aftercare, ensured them a place in a maternity hospital, saw to their medical treatment, and attempted to effect reconciliation between the woman and her family. They also tried, where possible, to make putative fathers pay maintenance for their children, often taking men to court. Moreover, the Committee also trained Glasgow University students in social work. The principle aim of this organization was to assist the young women to keep and wean their babies for at least six months in the interest of the child’s welfare.^62^

The SNSPCC also began to take a keener interest in what they called “unwanted children,” illegitimate children who “in most cases have a poor chance in life and … are very apt to be underfed.” Moreover, they lamented that the mothers of these children often had to go out to work, “thus depriving the child of the care and close attention that only a mother can give.” The SNSPCC also called for changes in legislation to deal with fathers of illegitimate children who persistently refused to contribute to the support of their children and identified this as a form of child neglect.^63^

This is in marked contrast to the prewar preference in Scotland which had been to have illegitimate children boarded out, a uniquely Scottish system in which children were placed almost exclusively with two-parent families paid to accept them. Macdonald argues that officials believed that by boarding out children within nuclear family households—the preferred stable family unit comprising parents with their own children—they would break the cycle of poverty among destitute children.^64^ As was the case in England and Ireland, many unmarried mothers were also institutionalized in poorhouses, hospitals, and asylums, some under the 1913 Mental Deficiency Act. This Act allowed officials to determine the fate of unmarried mothers and destitute and homeless married women. Because of their condition as paupers, these women could be classified as mental defectives if they applied for parish relief while pregnant or at the time when the baby was born. Parochial boards were also urged to refuse unmarried women outdoor relief.^65^

After the war, it was argued that illegitimate children had increased in importance, not just out of a sense of humanity but also in the national interest. New discourses were being established which argued that it was in the best interest of the child not to be separated from its mother especially during the breast-feeding phase.^66^ The shift from informal philanthropy to the establishment of organizations sanctioned by the Scottish Office and Public Health Committees to do so indicates the development of a more professional social work service and also a relative change toward an acceptance of illegitimate children, if not unmarried mothers, as well as greater state intervention in matters relating to women and children. For example, the Women’s Help Committee may have acted as an intermediary for collecting aliment from the fathers of the babies whose mothers they housed but at no point did they allow contact between the unmarried mother and the father of the child, suggesting that they feared further “delinquent” behavior on the part of the young women they dealt with.^67^

However, babies, irrespective of parentage, were increasingly seen as national treasures in the face of population decline, war deaths, and increased foreign military and economic competition. Indeed, the National Council of Public Morals which initiated the Birth-Rate Commission Reconstruction Enquiry may have frowned upon illegitimacy, but they were also anxious about depopulation and the “serious deficiency of young life” which the “tremendous changes wrought by war” had caused.^68^ The SNSPCC went so far as to claim that the war had resulted in former class feelings having been, “broken down” with the result that there was realization that no one section of the community can suffer through being deprived of the necessities and amenities of life without all classes suffering.^69^ Apparently, this guaranteed that there was an “enhanced interest of all classes in the importance of the life of children and a desire to remove all causes of disease and trouble” from the life of children, irrespective of their class or parentage.^70^ Whether it did or not illegitimacy while not significantly rising did contribute to an overall rise in lone parents in postwar Scotland.

## War Widows and the State

The most obvious form of family dissolution brought about by the conflict was the result of war widowhood. Although it has been ascertained that the majority of servicemen were young single men, around a third of the men who served during the war were married.^71^ The number of widows increased as the death toll mounted, and Scotland had a higher per capita death rate than other areas of Britain because the level of enlistment was higher and due to the fact that Scottish soldiers were sent into battle first as shock troops. The overall death rate for the British army during four years of war was 11percent. Of the 557,000 Scots who enlisted in the services, 26.4 percent lost their lives, a mortality rate two-and-a-half times that of the rest of the British army.^72^ The result of this was that in the early twentieth century, the majority of lone mothers were widows.^73^

However, the number of war widows varied according to enlistment and conscription patterns and would have been higher had it not been for the fact that two-thirds of men killed in action were bachelors.^74^ In Edinburgh, there was less essential work that would keep men from service and enlistment from this city was relatively high. The number of widows aged between twenty and twenty-four years of age rose from 30 in 1911 to 105 in 1921. Widows aged twenty-five to twenty-nine years increased from 148 to 353 and widows aged between thirty and thirty-four years rose from 424 to 846. Widows aged thirty-five to thirty-nine years also increased from 769 in 1911 to 1,156 in 1921. In Glasgow, 23,156 children’s fathers were dead in 1921, 7.6 percent of all Glaswegian children. Glasgow was the most highly populated conurbation in Scotland and voluntary enlisted and conscription were mediated, thereby the wealth of well-paid war work and the essential services which kept key workers out of the military. Yet over one in fifty men from this city lost their lives during World War I.^75^ In Aberdeen, the figure for children whose fathers were dead was 8.5 percent and in Perth it was 8.2 percent. By 1921, nearly one-third of all Scottish children were the dependents of widowed parents. Although not all of these children were fatherless because of war, significant numbers were. In Britain, there were 239,000 widows and 393,000 children receiving war pensions in 1921.^76^ In Scotland, over 13,000 widows were in receipt of a war pension in 1925.^77^

War widows’ pensions were an important source of income for many women, especially those with large families to care for. The pensions were also a windfall for able-bodied widows with one dependent who were not entitled to apply for poor relief because they had only one child. However, like separation allowances, the receipt of war pensions created a paternalistic relationship between widows and the state in which the state acted as a proxy of the dead spouse. Before the war, widows tended to receive more sympathy than other lone mothers from parish councils and charity organizations, but many local officials had a low opinion of them. In 1913, it was suggested by Glasgow Parish Council that although they wanted “to protect widows,” if they were given “too much money” this was “not being kind” as excess income “was a temptation to that class of mothers.” Like the married women in receipt of separation allowances, there was the assumption that war widows would squander pensions on alcohol and amusements to the detriment of their children’s well-being.^78^

Nor did widows receive a pension in their own right, but rather it was through the sacrifice of their dead husband. This provided authorities with their justification to monitor the conduct of widows, especially their moral and sexual conduct. There is evidence to suggest that if a war widow had an illegitimate child, she forfeited her pension. For example, Govan Parish Council records describe how Bridget received a war pension when her husband died in action, but as a result of having two illegitimate children after his death she was reduced to applying for poor relief and living with her children in lodgings.^79^ Flora also lost her war widow’s pension because she had an illegitimate child; and she also ended up living in lodgings, dependent on poor relief.^80^

A widow who decided to marry was not allowed to keep her pension because it was not rightfully her money but rather a payment by proxy, from her husband. Thus, widows received what Glasgow parish called “a dowry,” a lump sum equivalent to two years payment of the pension.^81^ According to Glasgow Parish Council, these dowries opened women up to the attention and exploitation of “unscrupulous men” who married widows for their dowry and then deserted them leaving them destitute and reliant “on the rates.”^82^ In 1918, 1,416 widows married bachelors. This rose to 2,435 in 1922 but then fell off thereafter.^83^ Although the claims of officials may have been exaggerated, there is evidence in the Criminal Officers Reports of war widows being exploited by men. In 1920, 4.8 percent of the women who were deserted, and whose husbands had been traced and prosecuted by these officers, were war widows. For example, Thomas married a widow who had a war pension, then deserted her and their eight-month-old child, denying paternity. As a result, his wife was not offered outdoor relief but ended up in the poorhouse with the child. David also married a war widow and then deserted her becoming involved with another woman who became pregnant with his child.^84^

Those war widows who did remarry presumably did so for love and companionship and for financial support because war pensions left many women on or below the poverty line. The wife of a private, if she was aged between thirty-five and forty-five years and had eight children, was entitled to 30 shillings 6 pence. The maximum pension any widow could receive was 37 shillings 6 pence. This was not a substantial sum when compared to the average wage for British blue-collar workers which was just over 100 shillings in 1920.^85^ Widows over forty-five years of age received less and were limited to 15 shillings and remuneration for their children. Moreover, widows faced long delays in obtaining their pensions and were left economically as well as emotionally disadvantaged by the loss of their partner.^86^ Delays in receiving pensions may explain why Kilmarnock parish records show a considerable rise in the number of widows applying for relief. In the period 1911 to 1914, 33 percent of applicants were widowed women, by the mid-war period this had risen to over 44 percent, and between 1918 and 1921 they accounted for 45 percent of applicants.^87^

## Temporary Separation and the State

Other wives whose husbands were physically or mentally injured by war were affected too. Many ex-servicemen’s wives struggled because their husbands were in hospital or asylums. Roper maintains that one in four men who fought in World War I was wounded and that 40 percent of all serving soldiers were in receipt of a pension with 200,000 officially recognized as suffering from war-related nervous conditions. What is notable from the Table 3 is that although there were regional variations with cities like Glasgow, where reserved occupations would have reduced levels of conscription, rates of widowhood were generally higher by 1931 than they were in 1921, suggesting that the war injuries contributed to rising levels of female-headed households.

**Table 3.** Number and Percentage of Illegitimate Births in Scotland.

Year	Aberdeen	Dundee	Glasgow	Perth
1911	10.9	11.2	10.8	11.0
1921	11.6	12.0	11.0	11.9
1931	11.8	12.3	11.1	11.4

*Source:* HMSO, “Preliminary Report,” *Census of Scotland*, 1921 and 1931.

Wives of the physically and psychologically wounded ex-servicemen often had to work to supplement men’s war pensions, take on all household chores normally done by husbands and care for home and children as well as injured veterans.^88^ Moreover, the families of men injured in war did not always receive their entitlements. Proof had to be provided that an ex-soldier’s illness was due to, or aggravated by, active service and then a gratuity of £100 was bestowed. Long-term illness or frequent bouts of illness therefore pushed families into poverty as allowances were eaten up, further aggravating the strain which hospitalization could bring. Of all the women who were temporarily separated from spouses who applied for relief to Govan Parish Council, 20 percent were women whose husbands were either in hospital or in prison. Roper argues that the emotional damage caused by war showed itself in ex-soldiers’ home life. The trauma and brutalization of war also showed itself in a rising incidence of domestic abuse which often led to marriage breakdown.^89^ For example, Mary^90^ married her husband during the war but left him in 1921 due to his cruelty, taking their two children with her and was compelled to seek financial assistance from Glasgow parish.^91^

William Cochrane, a war veteran, was sent to an asylum after the SNSPCC and parish officials had visited the family home on a number of occasions at the behest of his wife. Seemingly, she and their children were terrified of him. On his return from the front, he frequently got intoxicated and when drunk he became extremely violent.^92^ Joseph Coleman, an ex-soldier, often got intoxicated and when drunk wrecked the family home. He too was also sent to an asylum.^93^ While Roper highlights how many ex-servicemen could not adjust to family life, Kovan argues that they could empathize with the helplessness and defenselessness of children, having experienced extreme vulnerability themselves during the conflict.^94^ The case of William McLean perhaps epitomizes the extent to which the war could influence men’s relations with their families. William was shot in the head during the conflict and contracted a form of epilepsy, but was discharged from hospital as cured. During the war, his wife’s conduct had resulted in the four children of the marriage being taken into care. On his demobilization, William came back to his wife and the children were returned to the family home. Shortly afterwards, the marriage broke down again and William’s wife deserted him. They were reunited on a number of occasions but in each case his wife left him. There are no details as to why she did so, but according to Glasgow parish officials, William’s wife was nothing more than a “common prostitute.” Whatever tensions led to William’s marriage breakdown, he tried to keep his family together and care for his children. However, he fell under the radar of parish council officials and the officers of the SNSPCC when he was reported for child neglect because of the unkempt appearance of his children. At this point, William, along with his lodger Thomas Hunt, was caring for the children. When investigated by parish officials, it was determined that the children, although rather untidy, were well clad and had sufficient bedding and food, but that the home was messy. William was visited again and it was clear from the report that the officials were aware that he and his lodger had made an effort to clean the home and children. Nevertheless, it was decided that the children should be put into care again. William, whom officials suggested still looked rather “mental,” agreed to this if the children could be placed in a Ministry of Pensions children’s home. This proved impossible because such institutions were for orphans only. The parish then insisted that the children be placed in St. Benedict’s Children’s Home in Rutherglen where they had been cared for during the war. However, the superintendent of St. Benedict’s would only take charge of the children under a court order which would place the children in care until they reached maturity and which would have denied William access to them. It seems that William had been “difficult” when he had visited the children before their release into his care. The decision to place the children in St. Benedict’s was taken on January 20, 1920. On January 22, William sought out his wife and found her in a public house. He then cut her throat and was charged with attempted murder. The children were taken into care the next day.^95^ William spent twelve months in prison after being convicted of attempted murder.^96^

Whether William had intended to plead with his wife to reconcile to stop the children being taken into care, or whether he blamed her for the situation, or alternatively he was still suffering from the psychological effects of war and the removal of his children pushed him over the edge is unclear. What is evident is that he cared for his children and that there was no real evidence of neglect in this case. In Scotland, there was a perception that lone fathers were incapable of looking after young children on their own. The children of men separated from their wives and widowers frequently had their children taken into care.^97^ The effects of marriage breakdown, which war contributed to, were not only felt by lone mothers and their children. However, some women were able to use wartime conditions to escape previously unhappy and abusive relationships. Others were less fortunate. Nearly one-third of the female applicants for relief to Govan Parish Council in 1921 who sought financial assistance because their husband was in prison were victims of wife assault.

## Family Desertion

Roper highlights how many of the men who fought and returned from World War I found it difficult to adjust to family life.^98^ This was certainly the case in postwar Scotland where significant demobilized soldiers refused to return to their family homes. In July 1919, Glasgow Parish Council claimed that they were receiving fifty applications for relief each month from the abandoned wives of demobilized soldiers and sailors.^99^ Officials and charity organizations, especially the SNSPCC continued to stress the destructive influence on families when they complained in 1921 about the “great evil of the times” by which they meant “the readiness of men to abandon their wives and children.”^100^

Historians have shown that women were more likely to be among those living in poverty and therefore to be recipients of poor relief, especially those who found themselves lone parents. Women in such circumstances experienced poverty due to the loss of the major breadwinner and their inability match men’s wages, earning only one-third to one half of the average man’s wage.^101^ In 1909, the Registrar General of Scotland claimed that the average yearly rate of applicants for poor relief per head of population was one in fifty. James R. Motion, Inspector of the Poor and the clerk to Glasgow Parish Council, contested this figure. He claimed that the statistics of poor relief were based on a “flashlight view” and that the rates of applications were as high as forty-one applicants for every thousand of the population in rural Sutherland and 31.2 per 1,000 population in Scotland’s larger cities. Moreover, women applied for poor relief in greater numbers than men. Applications from women represented 32.9 for every 1,000 women in Glasgow in contrast to male applications which stood at twenty-two applications for every 1,000 men in the population. Motion attributed more than half of the female applications to widowhood, but also important were a significant number of wives who were deserted and separated.^102^

Applications for relief by deserted wives fell sharply during the war, as separation allowances and the availability of work for women improved their economic prospects. Parish councils could also trace husbands who had deserted their wives if they enlisted in the army: Glasgow Parish Council traced 275 men who had abandoned their families through their enlistment in the British army and a further thirty-two who had joined the Dominions Expeditionary Forces. Most of the wives of these men subsequently received separation allowances.^103^ This was important for poorer women and a windfall for able-bodied wives with one child who had been deserted because, as was the case with widows, they were not entitled to parish relief if they fell on hard times.

Many husbands who had deserted their wives and had formed new relationships were also uncovered, as wives sought separation allowances. Men who had deserted their families were given an amnesty during the war against prosecution, but once detected they were expected to maintain their families.^104^ Those who had committed bigamy were often less fortunate. For example, Frank Duff had deserted his wife and family and bigamously married a “clerkess” from Perth, maintaining that he was a widower, before enlisting in the Scot’s Greys and being sent to the front. However, when his legal wife applied for a separation allowance Duff was investigated and prosecuted, receiving three months in prison. In a similar case, Thomas Kerr was already married when he wedded a woman from Troon before enlisting in the army. When she applied for a separation allowance, Kerr was revealed to be a bigamist and was arrested. He too received a three-month prison sentence. William Spence, a married man, was more fortunate when he bigamously married a domestic servant, passing himself off as a bachelor. He was admonished because he “could do more good at the front than in prison.”^105^ According to the Scottish Prison Commission, there was a significant increase in the number of individuals incarcerated for bigamy during the war years, but this was most likely a reflection of the ease by which men, who had deserted their wives and bigamously married another woman, could be traced through military service rather than an actual increase in bigamy.^106^

Table 4 highlights the ability of deserted wives to apply for separation allowances and the expansion of war work significantly reduced the number of wives who were compelled to seek poor relief and indicates the economic progress of wives during World War I.

**Table 4.** Applications for Relief by Deserted Wives to Glasgow Parish Council, 1913–1921.

Year	Number
1913	1,914
1914	1,951
1915	1,317
1916	840
1917	664
1918	564
1919	644
1920	1,168
1921	1,507

*Source:* Glasgow Regional Archive, T.PAR 1:33, Parish of Glasgow, Collection of Prints, November 1921.

In 1914, there were 1,914 applications for relief by deserted wives in Glasgow, but by 1918 this had fallen to 644 only to rise again in 1920 to 1,168 applications. A similar pattern was found in Kilmarnock, a textile town where work was available for women. Applications from deserted wives were at their highest in the period prior to the outbreak of war, dropping by over 60 percent during the hostilities.^107^ Throughout Scotland, the trend was toward a slight rise in the percentage of applications from deserted wives at the beginning of the war, as women awaited their separation allowances or husbands to be traced through enlistment. Applications then fell during the war.^108^

Before the war, parish councils had indicated that wife desertion and the family poverty that it caused was the result of men’s conduct, largely their unwillingness to work, their infidelity and drunkenness, although they did suggest that slovenly wives were also a source of family breakdown.^109^ In 1913, Glasgow Parish Council investigated the causes of desertion in the city. The inquiry looked into 400 cases of wife desertion. James R. Motion, Inspector of the Poor, argued that family desertion was the result of men’s drunkenness, criminality, immorality, and willful neglect in 85 percent of the cases they came into contact with. Apparently, only twenty-two wives were deserted due their own conduct.^110^ Nevertheless, like many of the wives who received separation allowances and the widows who were in receipt of war pensions, deserted wives were treated with extreme suspicion. Before the war, they were more likely than widows to be refused outdoor relief because of fears over what was called “desertion collusion.” This was the idea couples faked a marriage breakdown to defraud parish councils. Glasgow Parish Council housed deserted wives and their children in poorhouses who could not provide evidence of where the deserted husband had gone.^111^ Between December 1909 and May 1910, Glasgow Parish Council received 452 applications for assistance from deserted wives, 46 were given outdoor relief and 173 indoor relief.^112^ The Royal Commission of 1909, which looked at poor relief administration in Scotland, had recommended that except in special cases, outdoor relief should not be granted to deserted wives for a year; this was so that it could be ascertained that the women were indeed deserted and to ensure that husbands would not abandoned their responsibilities in the knowledge that the rate payer would maintain families outside of the poorhouse in their absence.^113^ This guaranteed that significant numbers of deserted wives were confined in poorhouses and separated from their children. Of the 7,106 children boarded out in Scotland in 1913, 11.2 percent were the children of deserted wives.^114^

Separation allowances and employment for women which resulted in declining numbers of deserted wives making applications for poor relief meant that those who did apply were more likely than before to receive outdoor relief during the war. Yet, this also resulted in the discourse on desertion changing. It came to focus more unsympathetically on the moral and sexual conduct of deserted wives, as it mapped onto discourses on soldiers’ wives and widows misspending their separation allowances and neglecting their children. Thus, marriage breakdown came to be attributed to women’s infidelity, drunkenness, and their lack of care for their homes and children.

In the postwar period, the rate of applications for relief from deserted wives began to rise again partly because of the retraction of separation allowances and the contraction of women’s war work that had masked the level of desertion. From a sample of just under 3,000 households from Govan Parish Council, Scotland’s third largest parish, for the years 1911 to 1929, applications from lone parents as a percentage of all household applications increased from 31.1 percent in 1911 to 35.9 percent in 1929 and those from deserted wives rose from 30.4 percent in 1911 to 37 percent by 1929.^115^ Excluding those men who migrated to England or emigrated overseas separating from their wives, according to the 1921 Scottish census enumerators in their *Preliminary Report* more than 20,000 women identified themselves as married than men did indicating levels of marriage breakdown and also the gendered approaches to separation where men seen themselves as again “unmarried” and women as still tied to their husbands.^116^

Officials attributed much of the increase in marriage breakdown to the effects of war. The SNSPCC saw an increase in the number of women approaching them because their husbands had either deserted them or were refusing to contribute to the family home. In 1919, the Executive stated “men and women are not ready to settle down to old grooves after demobilization” and that many men were neglecting their families by failing to maintain them.^117^

Glasgow Parish Council reiterated,

A large percentage of recently demobilised soldiers, who after their four years or so of active service and freedom from the worries incidental to married life in the poorer localities, appear to have become unsettled and discontented on their return and as our records show this leads to quarrels and they clear out. Unfortunately it is frequently the case that on demobilisation a number have discovered that their wives had been unfaithful. Some are prepared to forgive and forget for the sake of the children; others decline to resume cohabitation.^118^

Long separation and the unsettling effects of war and wartime infidelity were seen to have contributed to marriage breakdown. Parish officials also maintained that this was partly due to the ways in which soldiers’ wives had “succumbed to the craze for amusement to dispel their anxiety” which resulted in them losing “interest in their home and family.”^119^ Where “slovenly” was not seen as directly caused by a wife’s misconduct, it was seen to create difficulties in marriages in other ways. Apparently, the rising incidence of desertion was also caused by married men on active service being “bunked in decent families” in Britain during their training for service and this in turn highlighting the squalor of their own homes. Men seemingly attributed the tawdriness of their homes, which they were no longer prepared to accept, to their wives lack of household skills.^120^

Officials also claimed that,

As a result of the nerve racking experiences of the Front Line men are in many cases barely accountable now for their actions after the comparative freedom from domestic cares of army life, and are strongly disinclined to settle down to these domestic cares and worries. Many of this account, have left good homes and we are regularly discovering them living a life of gaiety, posing as single men, and also living with another woman.^121^

The unsettling effects of war and wartime infidelity other wartime factors were seen to have contributed to increased marriage breakdown. Gordon looked at the irregular marriages which were legal and unique to Scotland. She found that during World War I, irregular marriages increased as many couples formally registered their marriage to ensure that wives received separation allowances. Others, many of whom had cohabited as man and wife prior to the outbreak of war, opted for an irregular marriage during the war for speed and to avoid the cost of church weddings to guarantee their wives were financially secure.^122^ Nonetheless, local authorities identified these wartime unions as “ill considered” which they suggested led in many cases to marriage breakdown in the postwar period.^123^

The authorities did accept that housing shortages and the postwar inflationary pressures on standards of living were creating problems in marriages. However, they were less likely to attribute desertion to the rising levels of unemployment despite this corresponding with a surge in emigration in this period. There was a sharp decline in the rate of emigration from England and Wales after the First World War.^124^ During the period 1911–1915, Scotland lost on average 5 percent of its population through migration to England and emigration, particularly to the United States and Canada. Between 1921 and 1931, this rose to 8 percent when emigration from England and Wales had fallen to 0.5 percent. Although the Scottish rate of emigration fell in the 1930s, it remained at 4.5 percent, almost prewar levels.^125^

Before the war, emigration had been a major cause and consequence of desertion with poor law officials believing that the philosophy of the working-class men who emigrated toward their families was that of “out of sight out of mind.” Officials understood that not all men who emigrated did so to avoid family responsibilities, but nevertheless they argued that men who emigrated without their families spent the increased earnings they could command in the dominions and the United States on frivolity and debauchery. Others apparently abandoned their wives in favor of a new partner in the country in which they had settled.^126^ While prosecution cases show that there were men who behaved in this way, there was no consideration that emigration was a manifestation of an already broken-down marriage caused by the dislocating effects of war.^127^

Neither did rising levels of marriage breakdown diminish suspicion over deserted wives’ conduct or whether they were indeed deserted. However, deserted wives were less likely to be refused outdoor relief in the 1920s, especially if they had children. Poor law officials’ perceptions of whether a woman was respectable or not did nonetheless continue to determine how relief was dispensed.^128^ Moreover, by this point, more attention was expended in trying to trace and prosecute deserting husbands due to the rising costs of maintaining their families, especially in the economic climate of the 1920s and 1930.

Levine-Clark highlights how, owing to the fact that it was difficult to ensure men maintained their families, particularly those who emigrated, the costs of maintaining families left behind began to rise considerably in the early twentieth century, and especially after World War I. In Glasgow, the cost of maintaining the families of men who deserted them amounted to £15,091 from January to October 1920.^129^ This led to serious attempts to track down deserting husbands and after the war regulations were put in place, allowing poor law officials to prosecute men who evaded their family responsibilities. To this end, Scottish members of parliament and poor law officials were prominent in demands for the introduction of legislation that would create reciprocal relations across the Empire to help to trace men who abandoned their families. Legislation was passed effecting this change in England and Wales, but because of Scotland’s different legal and administrative system the legislation was not adopted as it was seen as unworkable.^130^

Poor law officials, however, continued to demand Scottish legislation that would allow them to pursue men who emigrated and abandoned their families. In this context, the deserted wife regained her status as victim of an unscrupulous husband and calls were made by the Incorporated Law Society, Scotland, to make desertion a criminal offence.^131^ Parish officials aimed where possible to effect reconciliation between husbands and wives and also to extract repayment of any relief given to wives in their husbands’ absence. Where this was not feasible, they called for the establishment of Penal Colonies where men could be “reformed” and would work to maintain their families.^132^ They also demanded greater use of the Children’s Act which would extend prison sentences incurred for desertion because child neglect would also be part of the charges brought forward against men. To this end, they did work with the SNSPCC.^133^ There were also calls for “drastic action” to prevent men leaving the country without their wives and children and parish officials effected this when they oversaw the assisted emigration schemes of the 1920s and 1930s.^134^

Within Scotland, there was also an attempt made to deal with the many men considered to have given up their employment rather than maintaining their families. As benefits for the unemployed expanded from the 1920s, parish officials increasingly made demands on these. The benefit system, whether a couple were living apart or not, was structured around the male breadwinner so that an absent husband received the dependents’ allowance and was expected to provide for his family, but many men simply absconded with the benefit.^135^

However, the costs of wife and family desertion were not the only concern of officials. Levine-Clark maintains that the state regulated “unmanly behavior” as part of their postwar attempts to return to prewar gender norms.^136^ There were also fears over the effects of female-headed households on children. As children came under the lens of the state, fears of delinquency due to absent fathers and the necessity of mothers undertaking paid work, or maternal deprivation, were expressed. These were not, as is often suggested, a product of the post–Second World War period.^137^ In 1913, James R. Motion, Inspector of Poor for Glasgow Parish Council argued that,

No children reared under sordid conditions can be expected to become respectable law-abiding citizens; the home life lays the foundations of the character in after years and it is to this one must look for the main causes operating to produce large numbers of criminals.^138^

By 1920, officials were maintaining that wife desertion had “grave consequences,” particularly where “young helpless children were concerned” because,

Without a father’s restraint and guidance, which the father is more qualified to give, and further, because of the struggle to provide for the children, the deserted mother has not the opportunity to give them a proper upbringing, they are likely to drift into undesirable surroundings and modes of living … Where children are concerned there can be no excuse as it is a father’s natural and lawful duty to provide.^139^

Yet many children paid the price of their fathers’ inability or unwillingness to provide for them. Significant numbers of children were either boarded out, sent to live with relatives, and adopted out or placed in poorhouses and orphanages. These children included the illegitimate children born to soldiers’ wives, children of deserted and widowed women and of unmarried mothers some of whom who may have married after the war had the conflict not taken such a high toll on young men’s lives. It also included the children of ex-servicemen whose marriage broke down due to the dislocating effects of war. These parents were either forced to place their children into care through poverty or shame or persuaded to do so in the “best interest of the child.” Of all the applications on behalf of a child in 1921 to Govan Parish Combination from our sample, the majority were children of lone parents although 6 percent were the product of wartime extramarital affairs. Forty-three percent percent of these children resided with extended family and 18 percent were either adopted or lived with a guardian; the others were boarded out or placed in an orphanage whether they were orphaned or not.^140^ It would seem that prewar ideas about distancing the child from its poverty-ridden environment were not altered by the experiences of war.

According to Winter, “the lost generation of young men” had a limited impact on British interwar demography and by implications—beyond the emotional scars—on family life too. He argues that because women married younger and therefore their fecundity was increased, celibacy did not increase and the birthrate was not as adversely affected as might have been expected. Moreover, marriage rates were sustained by greater geographical and social mobility in marriage partnerships and also because of a sharp decline in the rate of emigration which had been dominated by younger men in the prewar years.^141^ However, Scottish women did not marry younger, and emigration rates rose above prewar levels in the 1920s, but the rate of marriage was sustained. In Scotland, there were 31,844 marriages in 1911 and this increased to 44,060 marriages in 1919 before falling back to around 32,000 in the 1920s and 1930s.^142^ By contrast, the birthrate fell markedly: there were 121,850 births in 1911, peaking in 1920 at 136,546 by 1920 and falling so that by 1930s, the rate was around 88,000.^143^

The economic climate of postwar Scotland may have convinced some women to postpone childbirth or reduce family size but the latter should not be overestimated because religious, cultural, and economic impediments reduced access to methods of birth control in Scotland, more so than was the case in England.^144^ Historians have also highlighted how the British birthrate fell in the 1930s, as maternal mortality increased partly as a response to the increase in illegal abortions as women sought to limit their family size in the face of the adverse economic conditions wrought by the depression.^145^ Yet, the falling birthrate may also have been due to the effects of emigration. Before the war, emigration had been both a cause and a consequence of marriage breakdown and other things being equal, with marriage rates being maintained, the average age of marriage for women remaining unaltered, and high emigration rates being sustained, it is feasible to conclude that the birthrate was also affected by war widowhood and desertion rates.

Challenging Winter’s hypothesis that the war did not result in higher levels of celibacy, Holden highlights how he based his findings on a narrow concept of singleness, that being a consideration of spinsters and their marriage prospects. The increase in the “surplus” of women, which had existed before the war, meant that widows, especially those with young children, had to compete on unequal terms with young single women in search of marriage partners.^146^ The same could be said of deserted, separated, and divorced wives and of unmarried mothers making lone motherhood a more permanent feature of many women lives than it had been before World War I.

## Conclusion

This article has highlighted the short-term gains of women during World War I and the long-term consequences of war in the rise in female-headed households and gendered and intergenerational poverty. In doing so, it contributes to the growing historiography on the effects of war on family structure, family breakdown, and family experiences. The state created the very conditions they sought to alleviate in the postwar period through enlistment and conscription which left vast numbers of women as heads of households and undermined the nuclear family. However, during the war many wives benefited from separation allowances and plentiful work for women especially poorer women or those whose husbands squandered the family income. The price of this security however was greater state surveillance and demonizing discourses on the behavior of soldiers’ wives. Unmarried mothers also made gains during the war. Illegitimacy rates may have risen relative to a declining birthrate during the later war years, but fears over military and economic competition, the declining birthrates and the death toll of war military and economic competition, the declining birthrates and the death toll of war rehabilitated illegitimate children and led to more direct state intervention in the lives of these children and their mothers which, in turn, led to the increased professionalization of social work services. Some unmarried mothers were provided with accommodation and child care, and deserted wives benefited from separation allowances, wartime work, and the ability of officials to trace absconding husbands and ensure that maintenance was forthcoming. In the postwar years, they were also advantaged from a change in policy that ensured that “respectable” wives who had been deserted were more likely than was the case before the war to receive outdoor relief. However, the economic consequences of war and mass emigration caused high levels of desertion in the postwar years and had detrimental effects on women and children. Not only were there greater levels of poverty, but also the strains of this resulted in further family breakdown, as lone parents were driven or persuaded to have their children placed in what was considered a more respectable and stable family unit.

Widows also made gains during the war through war pensions, and widows’ pensions for all widows were extended in 1925 and again in 1929 offering some protection. However, these were subsistence payments and they were strictly monitored. Nor were pensions regarded as a widow’s in her own right but rather they were a payment from the state acting as a proxy of the dead spouse. Other women benefited too, especially those who experienced cruelty in their marriages. By 1938, cruelty was included in the grounds for divorce in Scotland, although the emphasis was always on reconciliation where possible. Women may have benefited from the conditions of war in the short term, but the gains that they accrued revolved around concerns over the declining birthrate, the poor physical state of wartime recruits, and military and economic competition, in other words, the state of the nation.

In the longer term, female single-headed households increased because of the cumulative effects of men abandoning their families, war widowhood, higher levels of separation, and divorce and unmarried mothers. Women and their children came under greater state regulation through the welfare system and this did not end with the war. Discourses on the effects of fatherless children and working mothers resulting in juvenile delinquency were pervasive. Lewis argues that lone mothers’ status was achieved rather than ascribed.^147^ This made it easier for officials to attribute the poverty they endured to the fact that they did not have a breadwinner with all the underlying implications that they may have done something to contribute or cause their “achieved status.” This allowed the state and officials who dealt with them to ignore the particular difficulties faced by lone mothers. This guaranteed that prewar ideas about breaking the cycle of poverty among destitute children by removing them from the care of lone mothers prevailed. Many lone mothers were either forced through poverty or shame or persuaded to give up their children after internalizing the discourse that it was in the best interest of their child to have it boarded out in a nuclear family household with the result that family breakdown was exacerbated. Other lone mothers and their children endured the rising levels of gendered and intergenerational poverty and continued state regulation through the benefit system. This was the hidden cost of World War I in Scotland.

## Notes

1. Glasgow Caledonia University, GB 1847, CHILDREN 1st (RSSPCC) Archive, Royal Scottish National Society for the Prevention of Cruelty to Children, Annual Reports, [henceforth SNSPPC Annual Report], SNSPCC, Annual Report, Report by the Executive Committee, for year ending 1919, 6.

2. P. Thane and T. Evans, *Sinners? Scroungers? Saints? Unmarried Motherhood in Twentieth-century England* (Oxford, UK: Oxford University Press, 2012); T. Evans, “The Other Woman and Her Child: Extra-marital Affairs and Illegitimacy in Twentieth-century Britain,” *Women’s History Review* 20, no. 1 (February 2011): 45–65; K. Kiernan, H. Land, and J. Lewis, *Lone Motherhood in Twentieth-century Britain: From Footnote to Front Page* (Oxford, UK: Oxford University Press, 1998).

3. His Majesty’s Stationery Office [henceforth HMSO], “Preliminary Report: Dependents,” *Preliminary Report of the Thirteenth Census of Scotland*, 1921, Volume 4. (Edinburgh, UK: HMSO, 1921).

4. See J. Greaves, “The First World War and Its Aftermath,” in *Twentieth-century Britain: Economic, Cultural and Social Change*, ed. Francesca Carnevali and Julie-Marie Strange (Upper Saddle River, NJ: Pearson Education, Harlow, 2007), 127–44, which provides a useful section on further reading. For Scotland, see J. Kerr, *Scotland and the Impact of the Great War 1914-1928* (Paisley, UK: Hodder Gibson, 2010); exceptions include Gerard De Groot, *Blighty: British Society in the Era of the Great War* (London, UK: Longman, 1996); J. Winter, *The Great War and the British People* (Basingstoke, UK: Macmillan, 1985); and C. M. M. Macdonald and E. W. MacFarlane, eds., *Scotland and the Great War* (East Lothian, UK: Tuckwell Press Ltd, 1999).

5. See E. Cameron, *Impaled Upon a Thistle: Scotland Since 1880* (Edinburgh, Scotland: Edinburgh University Press, 2010); I. McLean, *The Legend of Red Clydeside* (Edinburgh, Scotland: John Donald Publishers Ltd, 2000); A. McKinlay and R. J. Morris, eds., *The ILP on Clydeside 1893-1932: From Foundation to Disintegration* (Manchester, UK: Manchester University Press, 1991).

6. For chronology of the historiography of war, see J. Winter and A. Prost, *The Great War in History: Debates and Controversies 1914 to the Present* (Cambridge, UK: Cambridge University Press, 2005), 625. For women’s work, see G. Braybon and P. Summerfield, *Out of the Cage: Women’s Experiences in Two World Wars* (London, UK: Routledge, 1987); T. Royle, *The Flowers of the Forest: Scotland and the First World War* (Edinburgh, Scotland: Birlinn, 2006); L. Snodgrass and C. M. M. Macdonald, “The Role of Scottish Women in the Great War,” *History Scotland Magazine* 13, no. 4 (July/August 2013): 24–29.

7. S. Kinsley Kent, *Making Peace: The Reconstruction of Gender in Interwar Britain* (Princeton, NJ: Princeton University Press, 1993); S. Alexander, “Men’s Fears and Women’s Work: Responses to Unemployment in London between the Wars’,” *Gender and History* 12, no. 2 (2000): 201–425. For Scotland, see A. Hughes, *Gender and Political Identities in Scotland, 1919-1939* (Edinburgh, Scotland: Edinburgh University Press, 2010), 13–37. For a discussion that gives an overview of the debate over progress and the backlash model, see A. Bingham, “An Era of Domesticity?” *Histories of Women and Gender in Inter-war Britain,* *Cultural and Social History* 1, no. 2 (2004): 225–33.

8. S. Pedersen, “Gender, Welfare, and Citizenship in Britain during the Great War,” *The American* *Historical Review* 95, no. 4 (October 1990): 983–1006, 1002.

9. J. Lomas, ““Delicate Duties”: Issues of Class and Respectability in Government Policy towards the Wives and Widows of British Soldiers in the Era of the Great War,” *Women’s History Review* 9, no. 1 (March 2000): 123–47.

10. Ibid.

11. J. A. Melling, ““Wicked Women from Wigan and Other Tales”: Licentious Leisure and the Social Control of Working Class Women in Wigan and St. Helens, 1914–1930,” *North West Labour History* 24 (1999/2000): 30–43.

12. Hughes, *Gender and Political Identities*, 13–17.

13. Pedersen, “Gender, Welfare, and Citizenship,” 991, 1002.

14. Ibid and SNSPPC Annual Report, Executive Report for year ending 1914, 8.

15. SNSPPC Annual Report, Executive Report for year ending 1915, 8.

16. Pedersen, “Gender, Welfare, and Citizenship,” 1001.

17. D. Coetzee, “A Life and Death Decision: The Influence of Trends in Fertility, Nuptuality and Family Economics on Voluntary Enlistment in Scotland, August 1914 to December 1915,” *Family and Community History* 8, no. 2 (November 2005): 79–89.

18. Pedersen, “Gender, Welfare, and Citizenship,” 985–98.

19. Ibid., 984–83.

20. Glasgow Regional Archive, [henceforth GRA], T.PAR 1.13-1.34, Collection of Miscellaneous Prints, [henceforth Prints], Parish of Glasgow, Statistical Report by the Inspector to the Parish Council for the 1/2 years ending May and November 1910–1922, [henceforth Statistical Report].

21. Ibid., November 1914.

22. SNSPCC Annual Report, Executive Report, 1914, 6.

23. Ibid.

24. ‘Relief of Distress: Glasgow’s Poor’, *Scotsman*, January 2, 1915.

25. Ibid.

26. Prints, Statistical Report for the 1/2 year ending November 1914.

27. Pedersen, “Gender, Welfare, and Citizenship,” 997.

28.
*Scotsman*, January 3, 1916. See also Melling, “Wicked Women from Wigan,” 30–43; Lomas, “Delicate Duties,” 123–47.

29. Prints, Statistical Reports 1/2 years ending May and November, 1914–1919.

30. Ibid.

31. J. H. Treble, “Unemployment in Glasgow 1903-10: Anatomy of a Crisis,” *Scottish Labour History Society* *Journal* 25 (1990): 8–39, especially p. 8 and p. 12 and Coetzee, “A Life and Death Decision,” 81–82.

32. See J. Melling, *Rent Strikes: People’s Struggle for Housing in West Scotland* (Edinburgh, Scotland: Tuckwell, 1983).

33. Coetzee, “A Life and Death Decision,” 81–82.

34. See J. Butt, “Working-class Housing in Glasgow 1900-1939,” in *Essays in Scottish Labour History: A Tribute to W. H. Warwick*, ed. I. MacDougall (Edinburgh, Scotland: John Donald Publications, 1978), 143–69 and Hughes, *Gender and Political Identities*, 67–70.

35. SNSPCC Annual Report, Executive Report, 1917, 9.

36. Prints, Statistical Report for the 1/2 years ending May and November 1914 and 1916.

37. A. Woollacott, ““Khaki Fever” and its Control: Gender, Class, Age and Sexual Morality on the British Homefront in the First World War,” *Journal of Contemporary History* 29 (April 1994): 325–47. See also P. Levine, ““Walking the Streets in a Way No Decent Woman Should”: Women Police in World War I,” *The Journal of Modern History* 66, no. 1 (March 1994): 34–78.

38. Registrar General of Scotland, Annual Report 1916, xiv.

39. Woollacott, “Khaki Fever,” 325–47.

40.
*Scotsman*, May 25, 1915 and October 6, 1916.

41. Woollacot, “Khaki Fever,” 335.

42.
*Scotsman*, October 6, 1916.

43. Ibid, May 15, 1915.

44. Ibid, May 26, 1915; October 6, 1916; and March 28, 1918.

45. Levine, “Walking the Streets,” 53.

46. Registrar General of Scotland, Annual Reports, 1911–1920.

47. Ibid., 1918, viii.

48. Ibid, 1915–17, 1915, vii.

49. Thane and Evans, *Sinners?*, 6.

50. Coetzee, “A Life or Death Decision,” 78.

51. Registrar General of Scotland Annual Report, 1915–17, 1916, xiii.

52. Coetzee, “A Life or Death Decision,” 78–79.

53. Registrar General of Scotland, Annual Report 1915, vii; A. Blaikie, “A Kind of Loving: Illegitimacy, Grandparents and the Rural Economy of North-East Scotland, 1750-1900,” *Scottish Economic and Social History* 14 (1994): 41–57.

54. Thane and Evans, *Sinners*? 2 and Evans, “The Other Woman,” 45–65, especially 48.

55. Thane and Evans, *Sinners?* 13.

56.
*Scotsman*, April 21, 1921.

57. Ibid.

58. T.PAR. 1.33, Prints, Glasgow Committee for the Care of Unmarried Mothers and their Children, Report, 1917.

59.
*Scotsman*, March 18, 1919.

60. For England, see P. Thane, “Unmarried Motherhood in Twentieth-century England,” *Women’s History Review* 20, no. 1 (2011): 11–29, 12.

61. Glasgow University Archive, GB 0248 DC260/5, Women’s Help Committee, ‘Pamphlet’, no date and ‘Warden’s Report’, Annual Report, 1925–1926, 3.

62. Ibid., Warden’s Report, 2.

63. SSNPCC Annual Report, Report by the Executive Committee, 1919, 11; and Report by the Executive Committee 1920, 11–12.

64. H. J. Macdonald, “Boarding-out and the Scottish Poor Law, 1845-1914,” *Scottish Historical Review* 75, no. 2 (October 1996): 197–220.

65. T-PAR 1:15, Prints, Dr John Macpherson, Senior Medical Commissioner in Lunacy in Scotland, “The Administration by Parish Councils of their Powers and Duties under the Mental Deficiency And Lunacy Amendment Act, 1913.” See also A. Blaikie, “Infant Survival Chances, Unmarried Motherhood and Domestic Arrangements in Rural Scotland, 1845-1945,” accessed September 15, 2013, http://www.localpopulationstudies.org.uk/PDF/LPS60/LPS60_1998_34-46.pdf; and for England, K. Holden, *The Shadow of Marriage: Singleness In England 1914-1960* (Manchester, UK: Manchester University Press, 2007), 115.

66.
*Scotsman*, March 18, 1919; Holden, *The Shadow of Marriage*, 117.

67. Women’s Help Committee, Pamphlet, n.d.

68.
*Scotsman*, April 22, 1918. See also GRA, T-PAR 1: 11, Prints, “Memorandum, Administration of the Poor Law,” 1913.

69. SNPCC Annual Report, Report by the Executive Committee, 1919, 8–9.

70. Ibid.

71. M. Roper, *The Secret Battle Emotional Survival in the Great War* (Manchester, UK: Manchester University Press, 2009), 5; Coetzee, “A Life and Death Decision,” 79–89.

72.
*Herald*, October 11, 2012.

73. Kiernan, Land, and Lewis, *Lone Motherhood*, 4.

74. Roper, *The Secret Battle*, 5.

75. www.educationscotland.gov.uk/higherscottishhistory/impactofthegreatwar, accessed September 16, 2013.

76. Pedersen, “Gender, Welfare, and Citizenship,” 1005.

77. Hansard (House of Commons), “Scotland Beneficiaries,” March 26, 1925, col. 597.

78. Lomas, “Delicate Duties,” 123–47.

79. D-Hew, 17/230–17/338999: Govan Parish Combination, Applications for Relief, 1921, Case Number, 17858208911. Surnames are not used to protect the identity of the applicants.

80. Ibid., Case no. 17/860/209817.

81. GRA, D-Hew, 33.2 Criminal Officers Report, 1920, Vol. XVII, [henceforth, Criminal Officers Report].

82. Ibid.

83. Registrar General of Scotland, Annual Reports, 1918, cxi and 1921, cvii.

84. Criminal Officers Report, 1920, Case Numbers 124833 and A66212, respectively.

85.
*Scotsman*, March 1, 1917 and A. Ritschl and S. Broadberry, “Real Wages, Productivity and Unemployment in Britain and Germany during the 1920s,” *Explorations in Economic History* 32 (1995): 327–49.

86. See J. Lomas, ““So I Married Again”: Letters from British Widows of the First and Second World Wars,” *History Workshop* 38, no. 1 (October 1994): 218–27.

87. Series CO3/42 & Dist 17/3, Kilmarnock Parish Council Applications for Relief, 1911-23.

88. Roper, *The Secret Battle*, 4, 289.

89. Ibid., 5–6, 294. See also E. Nelson, “Victims of War: The First World War, Returned Soldiers and Understandings of Domestic Violence in Australia,” *Journal of* *Women’s History* 19, no. 4 (2000): 83–106.

90. Forenames are used in certain cases to comply with access restrictions regarding the Data Protection Act 1998.

91. D-Hew, 17/230–17/338999, Govan Parish Combination, 1921, Case no. 17863311484.

92. GRA, D-Hew 33.1, Criminal Officers and Female Inspector’s Reports, 1919-1920, Vol. X.

93. Ibid.

94. Roper, *The Secret Battle*, 5–6, 294; S. Koven, “Remembering and Disremembering: Crippled Children, Wounded Soldiers and the Great War in Great Britain,” *The American Historical Review* 99, no. 4 (October 1994): 1167–202.

95. Criminal Officers and Female Inspector’s Reports, 1919–1920, Vol. X.

96. National Records of Scotland, High Court of Justiciary Processes, JC26/1920/58 William McLean, May 1920.

97. See L. Abrams, *The Orphan Country: Children of Scotland’s Broken Homes from 1845 to the Present Day* (Edinburgh, Scotland: John Donald Publishers, 1998), 84–87.

98. Roper, *The Secret Battle*, 5–6, 294. See also J. Bourke, *Dismembering the Male: Men’s Bodies, Britain and the Great War* (London, UK: Reaktion Books Ltd, 1996); D. Cohen, *The War Came Home: Disabled Veterans in Britain and Germany, 1919-1939* (Berkeley: University of California Press, 2001).

99. Prints, Statistical Report for the 1/2 years ending November 1919 and *Scotsman*, July 4, 1919.

100.
*Scotsman*, April 21, 1921.

101. See, for example, J. Humphries, “Female Headed Households in Early Industrial Britain: The Vanguard of the Proletariat?” *Labour History Review* 63, no. 1 (Spring 1998): 31–65; P. Thane, “Women and the Poor Law in Victorian and Edwardian England,” *History Workshop* 6 (Autumn 1978): 29–51; W. M. Gordon, “The Demographics of Scottish Poverty: Paisley’s Applicants of Relief 1861-1871,” *Journal of Scottish Historical* *Studies* 30, no. 1: 25–42; A. Clark, “The New Poor Law and the Breadwinning Wage: Contrasting Assumptions,” *Journal of Social History* 24, no. 2 (Winter 2000): 261–82.

102. T.PAR 1:17, Prints, “Poor Law Reform,” 1913.

103. T.PAR 1:31 Prints, “Examination of Applications and Prosecutions for Wife Desertion during the War Period,” 1919 and *Scotsman*, January 2, 1915 and January 3, 1916.

104. Prints, Statistical Report for the 1/2 years ending November 1914.

105.
*Scotsman*, February 19, July 27, and May 15, 1915.

106. House of Commons, Parliamentary Papers, *Annual Report of the Prison Commissions for Scotland* for the year ending 1916.

107. Series CO3/42 & Dist 17/3, Kilmarnock Parish Council Applications for Relief, 1911–23.

108. See O. Anderson, “Emigration and Marriage Break-up in Mid-Victorian England,” *The Economic History Review, New Series* 50, no. 1 (February 1997): 104–9 and J. C. Colcord, *Broken Homes a Study of Family Desertion and its Social Treatment* (New York: Russell Sage Foundation, 1919), at Project Gutenberg, accessed 11 February 2014, http://www.gutenberg.org/files/15420/15420-h/15420-h.htm.

109. T.PAR 1:13, Prints, “Desertion Causes, Supplementary Memorandum,” 1909 and *Scotsman*, 1909.

110.
*Scotsman*, March 6, 1913.

111. Prints, Statistical Report for the 1/2 years ending November 15, 1909.

112. Ibid., May 1910.

113. HMSO, *Royal Commission on the Poor Laws and Relief of Distress: Report on Scotland* (London, UK: HMSO, 1909), 114.

114. T.PAR 1:20 “Prints, The Children under the Poor Law,” 1913.

115. D-Hew, 17/230–17/338999, Govan Parish Combination Applications for Relief, 1911–1929.

116. HMSO, “Preliminary Report: Dependents,” *Census of Scotland*, 1921, Volume 4.

117. SNSPCC, Annual Report, Report by the Executive Committee, for year ending 1919, 6.

118. Prints, Statistical Report for the 1/2 years ending May 1919.

119. “Wife and Family Desertion: Causes,” Criminal Officers Report, 1920.

120. Ibid.

121. Ibid.

122. E. Gordon, “Irregular Marriage: Myth and Reality,” *Journal of Social History* 47, no. 2 (2013): 507.

123. “Wife and Family Desertion: Causes,” Criminal Officers Report, 1920.

124. Winter, *Great War and the British People*, 255–73.

125. James Gray Kyd, *Population Statistics* (Edinburgh, Scotland, 1952), accessed November 1, 2013, http://www.gro-scotland.gov.uk/files2/the-census/Webster_final.pdf.

126. T.PAR 1:18, Prints, “Wife and Family Desertion: Emigration as a Contributory Factor,” 1910.

127.
*Scotsman*, December 29, 1920; Criminal Officers Report, 1920.

128. “Wife and Family Desertion: Causes,” Criminal Officers Report, 1920 and Greenock Parish Annual Report “Rules of Relief,” May 1923.

129. Prints, Statistical Accounts for the years ending May and November 1920.

130. M. Levine-Clark, “From Relief to “Justice and Protection”: The Maintenance of Deserted Wives, British Masculinity and Imperial Citizenship, 1870–1920,” *Gender and History* 22, no. 2 (2010): 302–21. See also Evans, “The Other Woman,” 48 and *Scotsman*, April 4, 1914.

131.
*Scotsman*, October 4, 1922.

132. Criminal Officers Report, 1920 and Statistical Accounts for the years ending May and November 1920, 1921, and 1921.

133. Ibid.

134. Greenock Parish Annual Report, “Relief Committee Report,” February 15, 1928.

135. Levine-Clark, “From Relief to “Justice and Protection”,” 302–21.

136.
*Scotsman*, October 8, 1920; Levine-Clark, “From Relief to “Justice and Protection,” 315.

137. Criminal Officers Report, 1920.

138.
*Scotsman*, March 6, 1913.

139. “Apprehensions and Prosecutions, Supplementary Report,” Criminal Officers Report, 1920.

140. Abrams, *The Orphan Country*, 11–16, 88.

141. Winter, *Great War and the British People*, 255–73.

142. General Registrar for Scotland, *Time Series Data: Births, Deaths, and Marriages*, accessed November 1, 2013, http://www.gro-scotland.gov.uk/statistics/theme/vital-events/births/time-series.html.

143. General Registrar for Scotland, *Time Series Data*, accessed November 1, 2013, http://www.gro-scotland.gov.uk/statistics/theme/vital-events/births/time-series.html.

144. Hughes, *Gender and Political Identities*, 115–25.

145. See W. Secombe, “Starting to Stop: Working-Class Fertility Decline in Britain”, *Past and Present* 126, no. 1 (1990)” 126, 151–88 and especially T. McIntosh, ““An Abortion City”: Maternal Mortality, Abortion and Birth Control in Sheffield, 1920-1940,” *Medical History* 44 (2000): 75.

146. Holden, *The Shadow of Marriage*, 1–24.

147. Lewis, from the footnote to front page, 6.

